# Expression and Evaluation of a Novel PPRV Nanoparticle Antigen Based on Ferritin Self-Assembling Technology

**DOI:** 10.3390/pharmaceutics14091902

**Published:** 2022-09-08

**Authors:** Dan Li, Haozhi Song, Jialei Li, Xingjian Liu, Xintao Gao, Tong Wu, Zhifang Zhang, Yinü Li

**Affiliations:** Biotechnology Research Institute, Chinese Academy of Agricultural Sciences, Beijing 100081, China

**Keywords:** Peste des Petits Ruminants, hemagglutinin, ferritin nanoparticle, *Escherichia coli* prokaryotic expression system, silkworm baculovirus expression vector system

## Abstract

Peste des Petits Ruminants (PPR) is a highly pathogenic disease that is classified as a World Organization for Animal Health (OIE)-listed disease. PPRV mainly infects small ruminants such as goats and sheep. In view of the global and high pathogenicity of PPRV, in this study, we proposed a novel nanoparticle vaccine strategy based on ferritin (Fe) self-assembly technology. Using *Helicobacter pylori* (*H. pylori*) ferritin as an antigen delivery vector, a PPRV hemagglutinin (H) protein was fused with ferritin and then expressed and purified in both *Escherichia coli* (*E. coli*) and silkworm baculovirus expression systems. Subsequently, the nanoparticle antigens’ expression level, immunogenicity and protective immune response were evaluated. Our results showed that the PPRV hemagglutinin–ferritin (H-Fe) protein was self-assembled in silkworms, while it was difficult to observe the correctly folded nanoparticle in *E. coli*. Meanwhile, the expression level of the H-Fe protein was higher than that of the H protein alone. Furthermore, the immunogenicity and protective immune response of H-Fe nanoparticle antigens expressed by silkworms were improved compared with the H antigen alone. Particularly, the protective immune response of H-Fe antigens expressed in *E. coli* did not change, as opposed to the H antigen, which was probably due to the incomplete nanoparticle structure in *E. coli*. This study indicated that the use of ferritin nanoparticles as antigen delivery carriers could increase the expression of antigen proteins and improve the immunogenicity and immune effect of antigens.

## 1. Introduction

Peste des Petits Ruminants (PPR), or ovine rinderpest, is an acute, severe and contagious disease caused by the Peste des Petits Ruminants Virus (PPRV). PPR mainly infects small ruminants such as goats and sheep and is characterized by fever, stomatitis, gastritis, diarrhea and pneumonia [1,2]. PPRV consists of a 15,948 bp non-segmented single-stranded RNA genome, which encodes six structural proteins and two non-structural proteins [3,4]. Among them, F and hemagglutinin (H) proteins are the most immunologically relevant determinants of protection against the measles virus, containing several T–cell epitopes and most of the neutralizing antibody epitopes [5,6,7]. Both F and H proteins induce host immune protection. The H protein stimulates a stronger humoral immune response and more neutralizing antibodies than the F protein [8,9]. Due to the high incidence of PPR, the mortality rate can reach almost 100% in severe cases [10], which poses a great threat to sustainable small-scale agriculture, especially small ruminant farming systems. The disease has spread to more than 70 countries, including Africa, the Near and Middle East and Asia. More than 1.7 billion sheep and goats are infected [11], which causes serious economic losses [12,13]. The PPR epidemic was first reported in Tibet, China, in 2007 and was effectively curbed by slaughtering infected animals, introducing vaccinations and imposing restrictions on animal transport in PPR-infected areas [14]. PPR subsequently re-emerged in Xinjiang, China, in December 2013 and spread rapidly to most parts of China in 2014. Subsequently, it was significantly brought under control throughout the country [15].

At present, PPRV control strategies depend on live-attenuated vaccines. The attenuated PPRV vaccines Nigeria 75/1 and Sungri 96 are the most widely used worldwide and are considered to be completely attenuated, safe and effective. These two vaccines also provide lifelong protection for immunized animals [15]. However, these live-attenuated vaccines have poor thermal stability and are often subject to harsh transport conditions, especially in subtropical regions. Live-attenuated vaccines also have the risk of reversing into virulent phenotypes and cannot distinguish infected animals from vaccinated animals (DIVA), which complicates control and regulatory measures [16]. Therefore, the development of a new vaccine with heat resistance, high efficiency and the ability to identify vaccine-immunized animals has become key to effectively eliminating the PPR epidemic.

Because they have a small immune doses, good biocompatibility and site-specific targeting, nanoparticles are increasingly used in the field of vaccine development. Among them, ferritin (Fe) is a type of iron storage protein which widely exists in organisms and is involved in a variety of physiological and pathological processes [17]. Ferritin consists of 24 subunits, each of which forms a trimer subunit, and eight trimers form the highly symmetrical octahedral cage structure that is characteristic of ferritin nanoparticles [18]. Ferritin nanoparticles exhibit remarkable thermal and chemical stability, meaning they can be used as drug delivery carriers and scaffolds to display exogenous peptides or proteins [18,19,20]. In 2006, New Century Pharmaceuticals, Inc. Fused and expressed the Tat peptide of HIV at the N-terminal of the ferritin L subunit. The expressed protein had ferritin nanostructures and could induce efficient immune responses. Since then, self-assembled ferritins have been increasingly used in vaccine development.

In the virus-like particle (VLP) assembly of PPRV, the co-expressions of M, H and N proteins have higher expression levels than that of a single protein, which can also induce the high-level production of specific antibodies and protective immune responses [21]. Compared with a VLP vaccine, in this study, we only fused the main antigen H protein of PPRV with *Helicobacter pylori* (*H. pylori*) ferritin. In this study, the hemagglutinin–ferritin (H-Fe) fusion sequence was constructed to be expressed and self-assembled in spherical nanoparticles in the *Escherichia coli* (*E. coli*) expression system and the silkworm baculovirus expression system. Subsequently, the immune effects of the H-Fe and H antigens were evaluated. Nanoparticle antigens expressed by silkworms induced BALB/c mice to produce more high-level specific antibodies than the antigen alone, which could be used to neutralize PPRV and induce a protective immune response. However, due to the incomplete folding of the fusion protein in *E. coli* expression, the H protein did not induce higher-level titer-neutralizing antibodies after ferritin assembly. In conclusion, ferritin can be used as an ideal antigen delivery carrier to improve the immunogenicity of H proteins and represents a new strategy for the development of PPR vaccines. The post-translational modification function of the silkworm baculovirus expression system could improve the self-assembly of the fusion protein so as to induce an improved immune effect. Compared with the *E. coli* expression system, this system is more suitable for use with ferritin self-assembly technology.

## 2. Materials and Methods

### 2.1. Cells, Viruses and Animals

The defective-rescue BmNPV BES reBmBac vector and the recombinant BmNPV transfer vector pBmPH (GenBank submission ID: 2426173) were constructed and maintained in our laboratory [22]. The pET-28a (+) vector was saved in our laboratory. BmN cells were preserved in our laboratory and cultured in TC100 insect cell culture medium (Applichem, Darmstadt, Germany) with 10% fetal bovine serum (FBS, Gibco, Waltham, MA, USA) at 27 °C. Vero cells were cultured with DMEM medium containing 10% fetal bovine serum (FBS) at 37 °C in a 5% CO_2_ incubator. The *E. coli* strains DH5α and Rosetta (DE3) were cultured in Luria–Bertani (LB) medium. The PPR Clone 9 virus strain was purchased from TECON Co., Ltd. (Urumqi, China), Specific-pathogen-free (SPF) BALB/c mice were obtained from Beijing Vital River Laboratory Animal Technology Co., Ltd. (Beijing, China).

### 2.2. Construction of Recombinant Vector

#### 2.2.1. Construction of Recombinant Vectors pET-28a-H-Fe and pET-28a-H

After codon preference optimization, the PPRV H protein (GenBank: AJE30413.1) and *H. pylori* ferritin (GenBank: NP_223316, 5-167aa, N19Q) sequences were synthesized using GenScript (Nanjing, China) with *Bam*HI and *Eco*RI restriction sites at the upstream and downstream, respectively. The H protein sequence was fused to the N-terminal of ferritin by a linker (Ser-Gly-Gly). The H-Fe and H fragments were amplified via fusion PCR (Phanta Max Super-Fidelity DNA Polymerase was purchased from Vazyme), and then the H-Fe and H protein sequences were inserted into the pet28a vector by *Bam*HI/*Eco*RI (FastDigest enzymes *Bam*HI and *Eco*RI were purchased from Thermo Scientific) digestion to construct the vectors pET-28a-H-Fe and pET-28a-H.

#### 2.2.2. Recombinant BmNPV Construction

As shown in Section 2.2.1, the H-Fe and H fragments were amplified via fusion PCR and cloned into the pVL1393 vector to obtain pVL1393-H-Fe and pVL1393-H. Then, they were cloned into the pBmPH vector to construct the transfer vector pBmPH-H-Fe. Then, the transfer vectors were co-transfected (VigoFect was purchased from Vigorous Biotechnology Beijing Co., Ltd., Beijing, China) with reBmBac into BmN cells. Finally, the recombinant BmNPV baculovirus reBm-H-Ferritin and reBm-H were obtained from the supernatant of co-transfected cells. The recombinant viruses reBm-H-Ferritin and reBm-H obtained via co-transfection were used as the starting stocks for mono plaque purification, and the highly expressed strains were selected for amplification, which were used as the parent strains of the virus for further experiments.

### 2.3. Expression and Purification of H-Fe and H Protein

In terms of *E. coli* expression, 1 μL pET-28a-H-Fe or pET-28a-H plasmids were added to 50 μL *E. coli* Rosetta DE3 competent cells on a sterile operating table. After 30 min on ice, the mixture was heat-shocked at 42 °C for 90 s, then left on ice for 1 min. Added 1 mL LB medium, shook at 37 °C for 1 h. 200 μL bacterial solution was evenly coated on the culture plate containing kana resistance and cultured for 8–12 h. Monoclonals were picked and cultured in liquid LB medium containing kana resistance at 37 °C for about 12 h. According to the proportion of 1%, the cultured bacterial solution was added to the new LB liquid medium for further culture. After optimization, 0.5 mM IPTG was used in 7 h of induction to obtain the expression product. The supernatant and precipitation were separated after the expression product was ultrasonically disrupted, and then, they were analyzed via SDS-PAGE. The fusion protein was then expressed and purified via Ni-NTA affinity purification. To assemble the H-Fe protein in the inclusion body into spherical nanostructures, the fusion protein was refolded in dialysis buffer (Fe^3+^ with an appropriate concentration) with a gradually decreased urea concentration (Dialysis buffer: 20 mM Tris-HCl (pH8.0), 10% sucrose, 0.6 mM Arg, 0.2 mM EDTA, and then urea with concentrations of 6 M, 4 M, 2 M, 1 M and 0.5 M was added respectively to prepare dialysate.). The target protein after Ni-NTA affinity purification was loaded into the 3.5 kDa dialysis bag.

In terms of silkworm expression, the recombinant virus was injected into the internodes of the dorsal abdomen of fifth instar silkworm larvae or pupae, and then rearing was continued at 25–27 °C and 65% humidity. After 108–120 h, larval hemolymphs or pupae expressing the protein of interest were harvested. Crude samples were diluted with PBS containing ascorbic acid and subjected to mild sonication and ultracentrifugation (35,000 rpm, 3 h) with 30% sucrose as the medium for crude purification. The white clear pellet deposited at the bottom of the tube was resuspended with PBS overnight at 4 °C.

### 2.4. Identification and Concentration Determination of H-Fe and H Protein

The purified H-Fe and H proteins were verified via Western blot [23]. The His monoclonal antibody or the mouse anti-PPRV H monoclonal antibody (Pirbright Institute, Surrey, UK) was used as the primary antibody, and the HRP-labeled goat anti-mouse IgG antibody was used as the secondary antibody. To verify the correctness of the purified protein, SDS-PAGE was performed. The gel strip containing the target protein was cut and placed in PBS buffer, and then sent to Shanghai luming biological technology co., Ltd. for analysis by LC-MS/MS. Nano-HPLC liquid phase system EASY-nLC1200 (Thermo Scientific, Waltham, MA, USA) was used for separation, and Q-Exactive mass spectrometer (Thermo Scientific) was used for mass spectrometry analysis.

A transmission electron microscope (TEM) (New Bio-TEM H-7500, HITACHI, Tokyo, Japan) was used to observe whether the self-assembly of nanoparticles was successful or not. The purified protein sample was negatively stained, and the steps were as follows: 10 μL of the suitable diluted sample was dropped onto the coated copper network and incubated for 5 min. After being washed three times with distilled water and dried, 5 μL of uranyl acetate was dropped for staining and then dried and observed under the TEM. Immunogold labeling was used to identify whether H proteins were present on the surfaces of the nanoparticles. The specific operation was as follows: 10 μL of protein sample was dropped onto the coated copper mesh, washed with distilled water and dried. Then, 10 μL of 1:50 diluted H monoclonal antibody was dropped onto the copper mesh, incubated for 15 min, washed again and dried. After that, 10 μL of 1:40 diluted goat anti-mouse IgG (full molecule) gold-10 nm antibody (goat anti-mouse gold particle labeled secondary antibody purchased from Sigma Aldrich) was dropped onto the mesh, incubated for 15 min, washed, dried and stained with uranium acetate under the electron microscope.

The H-Fe and H proteins expressed in *E. coli* were purified via Ni-NTA affinity purification and quantified using the BCA Protein Assay kit [24]. We collected the expressed bacteria and washed twice with sterile water. We then added 20 mL of lysis buffer to the bacteria, suspended it and lysed on ice for 30 min. The bacteria were ultrasonically disrupted according to the procedure of breaking for 3 s and stopping for 5 s, 10 times for each group, and 3 groups in total. Then, the precipitate was collected by centrifugation, and the precipitate was washed with 20 mL of inclusion body washing solution I and II in turn. Centrifugation occurred at 12,000 rpm for 10 min, supernatant was discarded, precipitate was suspended with urea NTA0 Buffer and dissolved overnight at 4 °C. After centrifugation at 12,000 rpm for 10 min, the supernatant was filtered by a 0.45 μm filter and then loaded into a nickel gravitational column. An NTA-urea buffer with imidazole concentrations of 50 mM, 100 mM, 250 mM and 500 mM was used for elution, and the eluent was collected for SDS-PAGE detection.

ELISA was performed to detect the expression levels of the H-Fe and H proteins in silkworms, while the expressed and purified H protein was set as a standard substance. The serially diluted H protein expressed and purified by *E. coli* was coated in a 96-well plate, while, at the same time, the protein to be tested expressed by the silkworm was diluted 5-fold with PBS, sonicated and centrifuged, after which the supernatant was taken and made a 2-fold dilution from 100-fold with coating buffer. We then took 100 µL to coat on the microtiter plate and blocked with 5% skimmed milk powder for 3 h and washed four times with PBST. The mouse-derived anti-H monoclonal antibody (prepared by our laboratory) was used as the primary antibody, diluted 200 times with antibody diluent, and added to a 96-well plate at 100 μL/well, incubated for 1.5 h, and then washed four times with PBST. HRP-labeled goat anti-mouse IgG antibody (ZSGB-BIO, Beijing, China) was used as the secondary antibody, diluted 5000 times with antibody diluent, incubated for 1.5 h, and washed four times with PBST. After coloration with OPD, the absorbance was detected at 492 nm. The standard curve was made according to the values measured by the standard substance, and then the expression levels of H-Fe and H protein expressed in silkworms were calculated.

### 2.5. Immunizations

Six-week-old BALB/c mice (*n* = 70) were randomly divided into six groups (*n* = 10; H-Fe (*E. coli* or silkworm), H (*E. coli* or silkworm), Luciferase (Luc), PBS and adjuvant groups). The antigens for the H-Fe and H groups were prepared by mixing 25 μg of H-Fe protein or an equimolar dose of H protein with a 15% Montanide GEL adjuvant (SEPPIC, Courbevoie, France). In the Luc injection group, mice were immunized with Luc expressed in silkworm using the same method as those mixed with the adjuvant. The Luc injection group, PBS group and adjuvant alone injection group were set as the control groups. Subsequently, all mice were multiple points intraperitoneally injected with corresponding antigen (H-Fe: 25 μg/100 μL; H: 20 μg/100 μL), injected with 100 μL of antigen per mouse. BALB/c mice were immunized in prime/boost mode on day 0 and day 21, respectively. Blood samples were collected from the orbital venous plexus on day 14 and 35 after prime-mode immunization. Mice were euthanized at week 6.

In the vaccine safety test group, mice were injected with 5-fold doses of H-Fe and H antigens. Serum was collected on day 0 and day 21. The mice were euthanized on day 21, dissected, and observed in appearance and tissue sections (CEICA-CM3050S). The obtained heart, liver, lung, kidney, muscle, stomach and spleen tissues were frozen in liquid nitrogen and stored in a refrigerator at −80 °C. Removed tissue, dropped OCT embedding agent to make it completely submerged, then put it into liquid nitrogen for 10–20 s. Subsequently, the tissues were sliced in a cryostat, with a thickness of 8 μm, and then placed on the slide. The cut samples were dried, fixed in 80% acetone solution for 15 min, and cleaned with distilled water. Dyed in hematoxylin solution for 5 min, rinsed with distilled water three times. The slides were placed in the differentiation solution for 10 s, and the absorbent paper was dried. The slides were put into the blue solution for 5 min and washed with distilled water for 2 min, and then dyed in eosin staining solution for 5 min and rinsed three times with distilled water. The slides were placed in 95% ethanol (3 s), 95% ethanol (3 s), 100% ethanol (3 s), 100% ethanol (1 min), dimethylbenzene (1 min) and dimethylbenzene (1 min) for dehydration and transparency, then observed under a microscope and photographed for the record.

### 2.6. Analysis of PPRV Hemagglutinin-Specific Antibody

The determination of H-specific IgG antibody in mouse serum was achieved using ELISA [25]. The purified H protein was diluted to 2 μg/mL with the coating solution, added to 96-well plates at 100 μL/well and incubated overnight at 4 °C. After washing three times with PBST, 300 μL of 5% skimmed milk powder blocking buffer was added to each well and blocked in a 37 °C incubator for 3 h. The serum of the mice used in the test was firstly diluted 100 times, then diluted according to a gradient of 2 times, and then added to the 96-well plates at 100 μL/well, with three replicates in each gradient. Subsequently, the HRP-labeled goat anti-mouse IgG secondary antibody (diluted with PBST containing 1% skimmed milk powder at 1:5000) (Bioss, Beijing, China) was incubated at 37 °C for 1 h. After OPD coloration in the dark, 50 μL/well 2MH_2_SO_4_ was added to terminate the reaction. The absorbance at 492 nm was measured on the microplate reader, and the *P*/*N* value was calculated.

### 2.7. Neutralization Assay

Vero cells (2 × 10^5^) (maintained in our laboratory) were cultured overnight in 96-well plates. After the complement was inactivated at 56 °C for 30 min, the serum to be tested was filtered for sterilization. The serum was diluted 10 times with serum-free DMEM medium and then diluted according to a gradient of 2 times. The continuously diluted serum was mixed with the same volume of PPRV strain (100 tissue culture infective dose 50% (TCID_50_)) and incubated at 37 °C for 1 h. The mix of serum and virus was added to the 96-well plates that contained Vero cells cultured in advance, with at least 8 replicate wells for each dilution. The virus control group and the Mock group were set, and the cells were further cultured in a 37 °C incubator. In this study, the onset of half of the virus group was observed as the starting point, and the total incidence of the virus group was the statistical node. The number of cytopathic effect holes was observed and recorded under a microscope, and the neutralization value was calculated using the Reed–Muench method [26].

### 2.8. Statistical Analysis

All statistical analyses were carried out with GraphPad Prism software. Differences between groups were analyzed using two-way ANOVA. *p* < 0.05 was considered significant. * *p* < 0.05; ** *p* < 0.01; *** *p* < 0.001; **** *p* < 0.0001.

## 3. Results

### 3.1. Preparation of H-Fe and H Antigen

**Figure 1:**
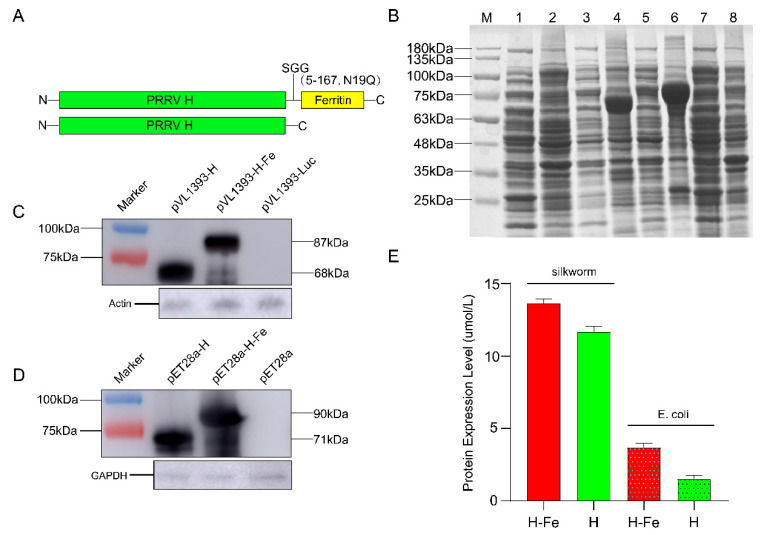
Construction and expression of the H-Fe and H proteins. (**A**) Molecular design maps of H-Fe and H sequences. (**B**) SDS-PAGE analysis of recombinant pET-28a-H-Fe and pET-28a-H expressed in *E. coli* Rosetta (DE3). Lane M, protein marker. Lane 1, pET-28a-H not induced and supernatant after sonication. Lane 2, pET-28a-H not induced and precipitated after sonication. Lane 3, pET-28a-H induced and supernatant after sonication. Lane 4, pET-28a-H induced and precipitated after sonication. Lane 5, pET-28a-H-Fe induced and supernatant after sonication. Lane 6, pET-28a-H-Fe induced and precipitated after sonication. Lane 7, pET-28a-H-Fe not induced and supernatant after sonication. Lane 8, pET-28a-H-Fe not induced and precipitated after sonication. (**C**) Western blot analysis of H-Fe and H recombinant proteins expressed in silkworm. The gray value of H-Fe and H protein bands were 32,076 and 45,489, respectively. (**D**) Western blot analysis of H-Fe and H recombinant proteins expressed in *E. coli*. The gray value of H-Fe and H protein bands were 33,518 and 49,246, respectively. (**E**) Detection of H-Fe and H protein expression level. Expression levels of silkworm-expressed proteins were measured by ELISA. Expression levels of *E. coli*-expressed proteins measured were by BCA.

Through restriction enzyme digestion and sequencing identification, pET-28a-H-Fe and pET-28a-H plasmids were successfully constructed. Subsequently, they were expressed in the *E. coli* Rosetta (DE3) strain and induced with 0.5 mM IPTG for 7 h. The expression products were detected via SDS-PAGE, as shown in Figure 1B. The results showed that there were obvious bands near 90 kDa and 71 kDa, which was consistent with the predicted fusion protein size, and it mainly existed in the form of inclusion bodies with less soluble expression. Subsequently, the fusion protein was expressed in large quantities, purified via Ni-NTA affinity purification, and then detected using the BCA Protein Assay kit. The results showed that the concentrations of the H-Fe and H proteins were 3.67 ± 0.31 μmol/L and 1.52 ± 0.25 μmol/L, respectively.

The H sequence was fused to the N-terminal of *H. pylori* ferritin by the “Ser-Gly-Gly (SGG)” linker (Figure 1A) [20]. The pVL1393-H-Fe and pVL1393-H plasmids were identified using enzyme digestion and sequencing, which proved the successful construction of plasmids. Then, well-growing cells were selected for co-transfection. The same batch of recombinant viruses obtained via the co-transfection of Luc plasmids was used as the quality control standard, and the luminescence detected was 2.3 × 10^6^ RLU, which proved that the process of recombinant viruses in this experiment was qualified. Then, the recombinant virus plaque was cloned and purified, and the high expression strain was selected for amplification and expression in healthy silkworms. The Luc recombinant virus underwent the same treatment and was then set as the control, and the luminescence detected was about 1.5 × 10^7^ RLU, indicating that the material silkworm was in good health and was not contaminated by wild viruses or other pathogens.

### 3.2. Western Blot Analysis and Mass Spectrometry Identification of H-Fe and H Protein

The H-Fe and H proteins expressed in *E. coli* were purified via Ni-NTA affinity purification (Figure S1). Western blot analysis was performed with the His monoclonal antibody as the primary antibody and HRP-labeled goat anti-mouse IgG as the secondary antibody. The results showed that the H-Fe and H proteins expressed in *E. coli* had clear bands near 90 kDa and 71 kDa, respectively, which were consistent with the predicted protein size, indicating the successful expression of the target protein (Figure 1D). Further identification via LC-MS/MS showed that the coverage rates of the H-Fe and H protein peptides reached 88% and 77%, respectively, and the molecular weights were consistent with the target protein, indicating that the H-Fe protein was successfully expressed.

The H-Fe and H proteins expressed in silkworms were subjected to ultrasonic fragmentation, centrifugation and filtration after appropriate dilution, followed by Western blot verification. The results of the Western blot analysis showed that the molecular weights of the H-Fe and H proteins were 87 kDa and 68 kDa, respectively. The results were consistent with the predicted molecular weights, indicating that the H-Fe and H proteins were successfully expressed in the silkworm expression system (Figure 1C).

### 3.3. Determination of H-Fe and H Recombinant Protein Expression Levels in Silkworms

In order to determine the content of recombinant protein expressed in silkworms, the expression levels of the fusion protein, H-Fe, and H were detected via ELISA, while the purified H protein expressed in *E. coli* was set as the standard substance. The results showed that the expression levels of the proteins H-Fe and H were 13.64 ± 0.30 μmol/L and 11.66 ± 0.41 μmol/L in the larval hemolymph, respectively (Figure 1E). The expression level of H-Fe nanoparticles was higher than that of the H protein alone, which proved that ferritin also effectively increases the expression level of the H protein in silkworms.

### 3.4. Electron Microscopic Observation of H-Fe and H Recombinant Protein

**Figure 2:**
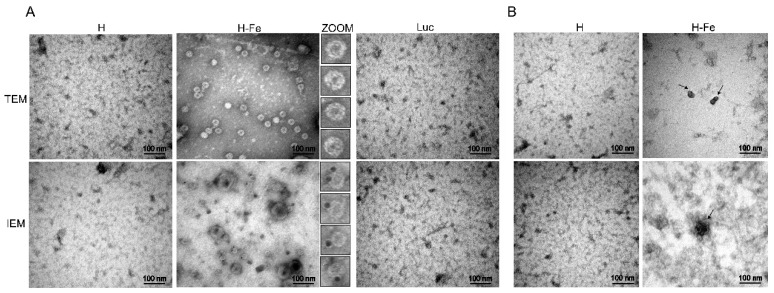
The TEM and IEM analyses of recombinant proteins H-Fe and H. (**A**) Electron microscopic observation of the fusion protein expressed in silkworms; Luc was expressed in silkworms as control. (**B**) Electron microscopic observation of fusion protein expressed in *E. coli*. TEM: transmission electron microscopy; IEM: immunoelectron microscopy. The scale bar is 100 nm.

The inclusion bodies in the H-Fe and H proteins expressed in *E. coli* were refolded via dialysis and observed using an electron microscope (Figure 2B). The results showed that a similar spherical structure was found in the H-Fe group, while a similar structure was not found in the H group. Further immunogold particle labeling of H proteins showed that H proteins were located on the surfaces of the spherical nanostructures. The results showed that H-Fe was assembled into spherical nanostructures via refolding, and the H protein was displayed on their surfaces. However, there were few nanoparticles in one field of vision, which may have been due to the low refolding efficiency, and most proteins were unable to fold in the correct way. More than 30 clearly visible nanoparticles could be observed from a silkworm expressed protein sample in one field of vision (4 × 10^5^ nm^2^), while only two spherical particles could be observed in the *E. coli* expression protein sample. These results showed that the silkworm eukaryotic expression system was more suitable for use with the self-assembly ferritin technology than the *E. coli* prokaryotic expression system.

The appropriate dilution of the crude purified sample after ultracentrifugation was carried out for sample preparation under electron microscopy. The TEM observation results (Figure 2A) showed that compared with the H proteins, clear spherical particles about 30 nm in size could be observed after negative staining on the H-Fe fusion proteins, which proved that the H-Fe fusion proteins could express and self-assemble ferritin efficiently in the physiological environment of silkworms. Furthermore, this also showed that compared with the prokaryotic expression system, the eukaryotic expression system has a post-translational modification function and is more conducive to the self-assembly of nanoparticles. Furthermore, for the immunogold particle labeling of the H proteins, the results from immunoelectron microscopy (IEM) showed that gold particles were attached on the surfaces of spherical ferritin particles, indicating that H protein was successfully displayed on the surface of the ferritin nanoparticles during the self-assembly process. This structure was similar to the natural virus which helped the host to recognize the antigen.

### 3.5. Safety Evaluation of H-Fe and H Antigens

**Figure 3:**
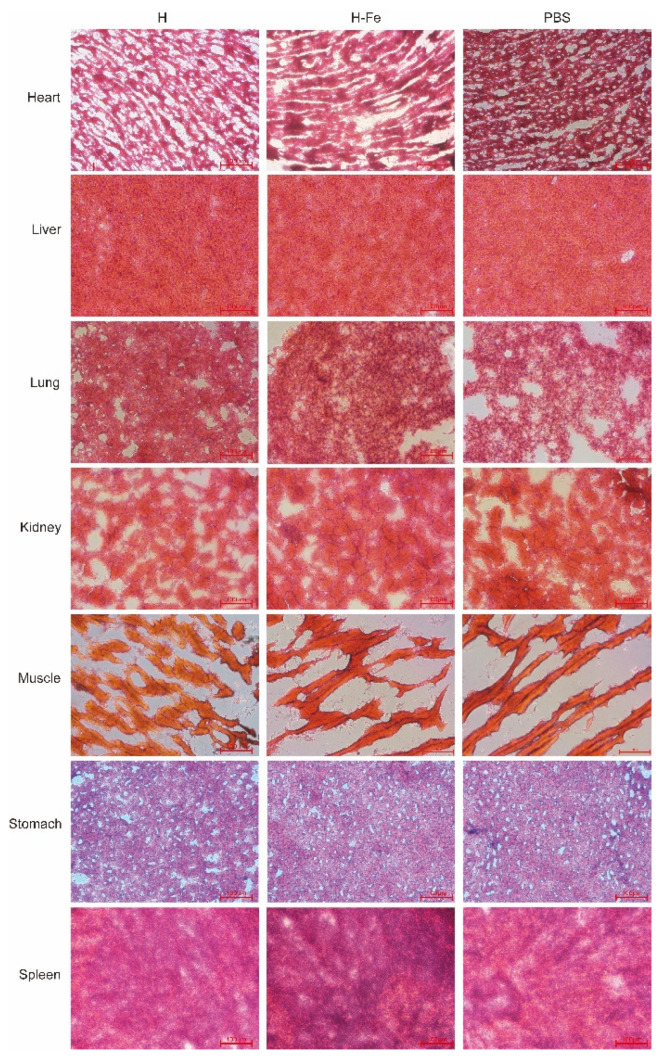
HE-stained sections of mice after immunization with antigens expressed in *E. coli*. The heart, liver, lung, kidney, muscle, stomach and spleen tissues were tested. The scale bar is 100 μm.

**Figure 4:**
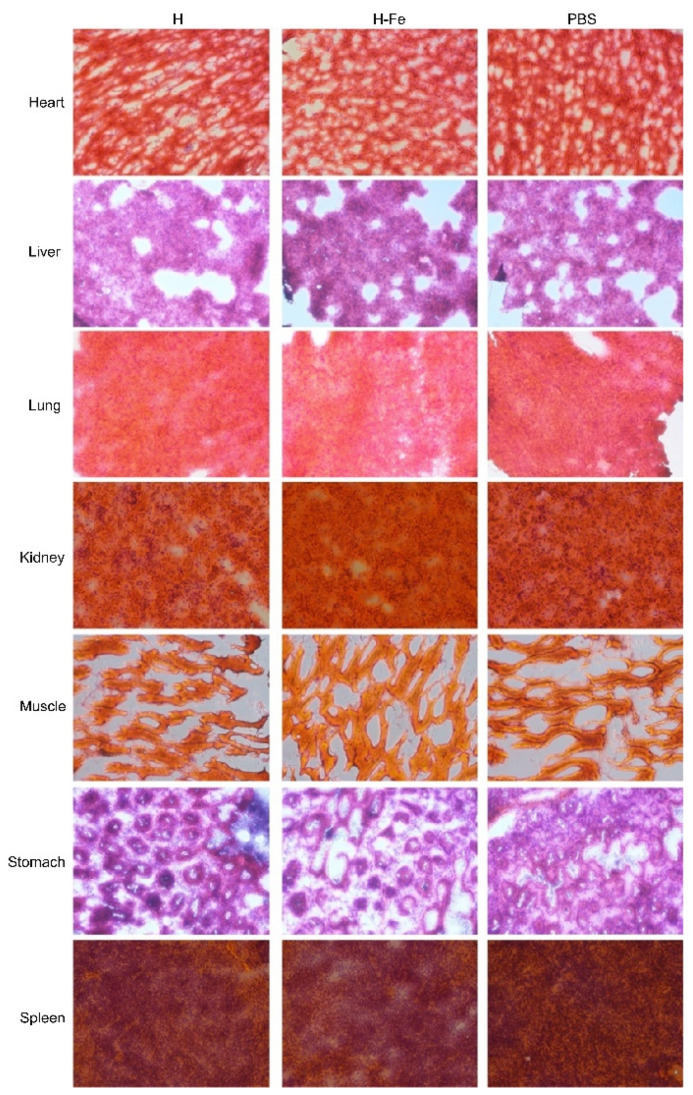
HE-stained sections of mice after immunization with antigens expressed in silkworms. The heart, liver, lung, kidney, muscle, stomach and spleen tissues were tested. The scale bar is 100 μm.

In order to investigate the safety of the H-Fe and H antigens expressed in *E. coli* and silkworms, we conducted a large-dose immunization test in mice. After high-dose immunization, no abnormal growth or physiological phenomena regarding aspects such as the weight, appearance, body temperature and behavior of mice were observed during growth. At the end of the experiment, after the euthanasia and anatomy of the experimental animals, no inflammation was observed in the abdominal cavity or in common organs. Seven tissues from the heart, liver, lung, kidney, muscle, stomach and spleen were sliced and observed in tissue slice experiments (Figure 3 (*E. coli*), Figure 4 (silkworm)). The results showed that there were no obvious histopathological changes in the dissected tissues of the mice compared to the experimental group and the control group, indicating that a 5-fold dose of antigens in order to immunize mice did not produce obvious side effects and was safe.

### 3.6. Detection of Antibody Titers in Serum of Mice Immunized with H-Fe and H Antigen

**Figure 5:**
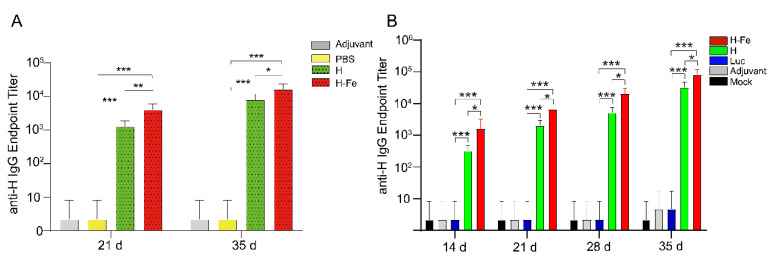
The detection of serum antibody titers of immunized mice in each group. (**A**) Antibody titer induced by antigen prepared by *E. coli* expression system in mice. Adjuvant: single-adjuvant immunization group; PBS: PBS injection group; H: H protein expressed in *E. coli* mixed-adjuvant immunization group; H-Fe: H-Fe protein expressed in *E. coli* mixed-adjuvant immunization group. (**B**) Antibody titer induced by antigen prepared by silkworm baculovirus expression system in mice. Adjuvant: single-adjuvant immunization group; Luc: Luc immune group; H: H protein expressed in silkworm mixed-adjuvant immunization group; H-Fe: H-Fe protein expressed in silkworm mixed-adjuvant immunization group. In the figure, ‘*’ indicates *p* < 0.05, ‘**’ indicates *p* < 0.01, and ‘***’ indicates *p* < 0.001.

To evaluate the immunogenicity of H-Fe and H, BALB/c mice were immunized with the H-Fe or H antigen twice at an interval of 3 weeks. Here, 25 ug of H-Fe protein or equal moles of the H protein were used per mouse. Serum was collected after the first immunization on days 14, 21, 28 and 35. Subsequently, the purified H protein expressed in *E. coli* was used as an antigen to detect the titer of the H-specific IgG antibody in mouse serum via ELISA. The results showed that with the prolongation of time, the antibody titer gradually increased. It was shown that on the day 21 after the first immunization, the specific IgG antibody titer induced by H-Fe nanoparticle antigens (*E. coli*) in mice was 4.3 ± 1.1 × 10^3^, which was shown to reach 1.7 ± 0.4 × 10^4^ on the day 35 after the first immunization (Figure 5). In addition, on the day 14 after the first immunization, the specific IgG antibody titer induced by H-Fe nanoparticle antigens (silkworm) in mice was 1.6 ± 1.2 × 10^3^, which was shown to reach 8.5 ± 3.0 × 10^4^ 35 days after the first immunization (Figure 5). Similarly, the specific IgG titer of H protein (*E. coli*) preparation antigens was 1.3 ± 0.3 × 10^3^ on the day 21 after the first immunization and 8.5 ± 2.1 × 10^3^ on the day 35 after the first immunization (Figure 5). The specific IgG titer of H protein (silkworm) preparation antigens was 3.3 ± 1.2 × 10^2^ on the day 14 after the first immunization and 3.4 ± 1.5 × 10^4^ on the day 35 after the first immunization (Figure 5). The levels of antibody titers induced by H-Fe nanoparticle antigens were significantly higher than those induced by H alone. The Luc group and adjuvant group did not produce H antibodies. These data showed that compared with the H antigens, the H-Fe antigens could induce mice to produce high-level serum antibodies earlier, and the H-Fe antigens could induce mice to produce higher levels of antibody titers than the H antigens, which proved that the self-assembly characteristics of ferritin enhanced the immunogenicity of the H protein.

### 3.7. Neutralization Test

**Figure 6:**
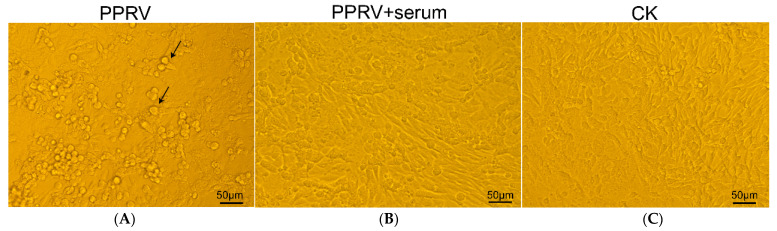
A schematic diagram of the cytopathic state in neutralization experiments. (**A**) PPRV virus control group; (**B**) Neutralizing antibodies completely inhibited PPRV; (**C**) CK. This study used 100 TCID_50_ PPRV virus solution. The CPE was characterized by rounding of cells, their detachment from the surface and syncytia formation. The virus-treated group had significant syncytial lesions, and the treated cells had no syncytia after the virus was completely inhibited by the appropriate dilution concentration of serum. The arrow points to the syncytium in the figure. The scale bar is 50 μm.

A neutralization test was carried out on serum antibodies produced by mice immunized with H-Fe and H antigens to evaluate the ability of serum samples to neutralize PPRV (Clone 9). The TCID_50_ of PPRV Clone 9 was 10^5^. The 35-day mouse serum was used for the neutralization test. The results showed that all of the cells in the virus control group showed cytopathic effects (CPE), while cells in the Mock infection group grew normally. In contrast to the normal cells, the cells infected with PPRV exhibited evident morphological changes, the CPE was characterized by rounding of cells, their detachment from the surface and syncytia formation. Figure 6 was shown as a schematic of the cytopathic state in neutralization experiments. In the only PPRV treatment group, there are syncytia appeared and clearly shown in the figure (Figure 6A); In the treatment group where the neutralizing antibody completely inhibited PPRV, there are no the lesions such as syncytia shown in the cells (Figure 6A), which was constant with the untreated control group (Figure 6C). No CPE was observed in the samples with high concentrations of H-Fe and H serum. When the H-Fe and H groups were diluted to a certain proportion, CPE began to appear in the cell pores. According to the calculation results from the Reed–Muench method, the titer of the neutralizing antibody induced in the H-Fe antigen (silkworm) group was higher than that of the H antigen (silkworm) group. The neutralizing titer in the H-Fe group was 1:135.5, and that in the H group was 1:68.1. The sera of mice immunized with Luc had no protective effect on viral infection, even in the highest concentration group. However, the neutralizing titers in the H-Fe and H (*E. coli*) groups were 30.20 and 28.18, respectively. The titers of the neutralizing antibodies induced by H-Fe and H antigens were basically the same, indicating that due to the limitations of the prokaryotic expression system, ferritin failed to effectively self-assemble, thus failing to induce an efficient protective immune response.

## 4. Discussion

At present, the control strategy for PPRV mainly depends on the administration of live-attenuated vaccines, but these live-attenuated vaccines have disadvantages; they have poor thermal stability, they are often subjected to harsh transportation conditions. In addition, it is difficult to distinguish animals vaccinated by live-attenuated vaccines and animals infected by PPRV. In response to this, an increasing number of PPRV genetic engineering vaccine strategies have emerged. The H protein has neuraminidase and hemagglutinin activity in Peste des Petits Ruminants virus [27], which is the main antigenic protein on the surface of PPRV and binds to the receptor on the surface of host cells. The H protein is an ideal immunogenic protein for inducing neutralizing antibodies in PPRV [8,28]. In previous studies, Bovine Herpesvirus-4 was used as a carrier of PPRV H antigens to protect sheep from PPRV attack [29]. Another study was carried out to express fusion protein (F) or hemagglutinin (H) using recombinant replication deficient-human adenovirus type 5 vector (Ad5) and inoculated sheep. This study showed that both recombinant adenovirus vaccines could induce PPRV- specific B-and T-cell responses, and neutralizing antibodies were detected in immunized sheep sera [16].

Nanoparticle vaccines are more likely to be captured and presented by dendritic cells (DCs) and macrophages than other types of vaccines that have been developed for PPRV [30]. In this study, a PPRV H-Fe nanoparticle antigen was prepared on the basis of ferritin nanoparticles, and in an experiment that involved immunizing mice, it was proved that it could cause strong immune responses. At the same time, it was shown that the induced antibodies could protect the host against the corresponding virus infection in vitro. Ferritin, as a nanoparticle, has a stable structure, high temperature resistance (70–80 °C) and resistance to various mutagens [31,32]. In addition, the use of silkworm expression systems to produce these nanoparticles is quite convenient. Compared to other nanoparticle vaccines containing artificial materials, all of the components of the nanoparticles that were constructed in this study were natural and highly safe. Furthermore, *H. pylori* ferritin and its antibodies are unlikely to be significantly toxic in vivo, based on the fact that no serious side effects were reported during the development or clinical evaluation of two *H. pylori* ferritin-based influenza nanoparticle vaccines (NCT03186781 and NCT03814720) [30]. Based on the advantages of ferritin, it is expected to become a good antigen- presenting carrier, which means a large-scale vaccination program for PPRV is possible.

In this study, we used the *E. coli* prokaryotic expression system and the silkworm eukaryotic expression system to prepare nanoparticle antigens. The Eukaryotic expression system is capable of post-translational modification and correct protein folding; therefore, it is more efficient and stable than the prokaryotic expression system. The baculovirus expression system (BES) is suitable for use regarding protein expression from eukaryotic sources. Many PPRV vaccines based on the BES have been developed [33]. In this study, the silkworm baculovirus expression system was used to prepare nanoparticle vaccines in silkworms. This system has a low production cost and enables a high expression level, and the effectiveness of nanoparticles is apparent, which means this system could be used to help control the transmission of PPRV in animals [34]. Compared with the eukaryotic expression system, the prokaryotic expression system is one of the widely used expression systems. As it enables rapid propagation, is easy to operate and enables the rapid production of *E. coli*, it has been used for the expression of multiple exogenous proteins [35]. However, as the prokaryotic expression system does not have post-translational modification functions such as glycosylation and phosphorylation, complex proteins cannot be correctly folded and thus cannot carry out their biological activities. Therefore, it must be determined how the expression level of exogenous proteins in the prokaryotic expression system can be increased and how the correct folding of exogenous proteins can be improved. In terms of the results from this study, compared with the H protein alone, the expression level of the H-Fe fusion protein in both the silkworm baculovirus expression system and the *E. coli* expression system was significantly increased, indicating that ferritin has the potential to increase the expression level of exogenous proteins.

Ferritin nanoparticles provide another strategy for the rational design of immunogens. Ferritin nanocages can enhance the immunogenicity of antigens by orderly displaying multiple antigens on their outer surfaces. This assumption is based on the fact that highly ordered repeats can induce stronger immune responses [20,36]. TEM analysis showed that the spherical structure with a diameter of about 30 nm was clearly observed. Meanwhile, IEM analysis showed that gold-labeled particles were attached to the surfaces of spherical particles, which proved that ferritin was self-assembled in the silkworm expression system, and the H protein was successfully displayed on the outer surface of ferritin. Similar nanoparticles were also observed in *E. coli* expression proteins, indicating that the expression of the H-Fe protein in *E. coli* could be assembled into higher structures in vitro. However, there were few nanoparticles in one field of vision, which may have been due to the low refolding efficiency, meaning most proteins could not fold in the correct way, which indirectly affected the titers of the antibodies produced via H-Fe fusion protein immunization in mice, especially the titer of the neutralizing antibody.

Nanoparticle vaccines have been successfully prepared by fusing antigenic proteins at the amino end of ferritin with connective peptides. In 2013, Kanekiyo et al. fused the HA protein of H1N1 subtype influenza virus with *H. pylori* ferritin to form a nanostructure with eight HA trimers on the surface, and compared with the inactivated trivalent influenza vaccine, the vaccine prepared with this fusion protein enhanced the antibody response by more than 10 times. Therefore, the antibodies induced with the nanoparticle vaccine could neutralize different H1N1 strains from 1934 to 2007, indicating that the ferritin self-assembly nanoparticle vaccine could induce strong humoral immunity and could also improve the efficacy and breadth of influenza virus immunity [20]. Another study showed that the VP6 protein of rotavirus was fused with the amino end of ferritin to be expressed in *E. coli* and self-assemble into a uniform spherical structure similar to ferritin. Mice were induced to produce high humoral and mucosal immunogenicity via oral administration [37]. Nanoparticle vaccines prepared through the fusion of antigenic proteins of HIV-1 [19], foot-and-mouth disease virus [38,39] and SARS-CoV-2 [30] with ferritin were shown to induce high levels of specific antibody titers and resist infection by the corresponding viruses. In this study, H-Fe nanoparticles expressed in silkworms were self-assembled into uniform spherical particles. After the antigen was mixed with the adjuvant, the BALB/c mice were immunized. Subsequently, 35 days after the first immunization, the specific IgG excited by the H-Fe nanoparticle antigens reached 85,333 ± 29,560.33, which was significantly higher than that of the H antigens alone. A further neutralization test showed that the neutralizing antibody induced by the H-Fe nanoparticle antigens was significantly higher than that induced by the H monomer antigens. These data showed that the H-Fe nanoparticle antigens could induce higher specific antibody levels than the H antigens alone, and the nanoparticle antigens induced higher neutralizing antibody levels, which enhanced the protective immune response. To achieve efficient presentation and interaction with cellular receptors, it is crucial for antigens to retain their structural stability and conformation. The successful self-assembly of nanoparticle antigens not only mimics the size and structure of pathogens but also facilitates the surface conjugation of antigens to promote interaction with immune cells [40]. However, it was found that the H-Fe fusion protein in the prokaryotic system has a very low correct folding rate, as it was difficult to observe correctly folded nanoparticle antigens. These results showed that fusion ferritin could produce higher specific antibody titers, and correctly folded nanoparticle antigens were more conducive to inducing the body to produce protective antibodies.

In summary, in our study we successfully confirmed that the H-Fe fusion protein can self-assemble into spherical nanoparticles and induce a higher immune response than H protein monomer antigens. The successful establishment of this method provides a new strategy for the design and production of PPRV vaccines. In addition, the synthesized nanoparticles were completely recombined, eliminating the possibility of producing potentially dangerous viruses in eggs or cell cultures and allowing antigen modification to enhance immunogenicity. In this study, BALB/c mice were used as animal models to study the feasibility of such nanoparticles, but in subsequent studies, we will explore the feasibility of this nanoparticle vaccine using large animals such as sheep and goats, etc.

## 5. Conclusions

In this study, we fused the PPRV hemagglutinin protein with *H. pylori* ferritin. The fusion protein was self-assembled in silkworm, and the H protein was also successfully displayed on the surfaces of ferritin nanoparticles. By evaluating the immunogenicity and effective immune response of antigens, we found that using ferritin nanoparticles as delivery carriers of antigens can significantly improve the immunogenicity and protective immune response of antigens. Fusion ferritin was shown to produce higher specific antibody titers, and it was shown correctly folded nanoparticle antigens were more helpful in inducing the body to produce protective antibodies. This study provides a concept for the development of a new PPRV nanoparticle vaccine and a potential vaccine strategy for the global elimination of PPRV.

## Data Availability

Data sharing is contained in this article.

## Supplementary Materials

The following supporting information can be downloaded at: https://www.mdpi.com/article/10.3390/pharmaceutics14091902/s1, Figure S1. SDS-PAGE analysis of purified samples of recombinant protein H and H-Fe. A. H protein purified sample; B. H-Fe protein purified sample.
