# Supplementary material for: Expression and Evaluation of a Novel PPRV Nanoparticle Antigen Based on Ferritin Self-Assembling Technology
Source: Pharmaceutics. 2022 Sep 8;14(9):1902. doi: 10.3390/pharmaceutics14091902 (PMC9500948; doi:10.3390/pharmaceutics14091902)
Supplement: Supplementary file 1 [file pharmaceutics-14-01902-s001.zip › Highlights.pdf]

Highlights

The fusion expression of PPRV Hemagglutinin and ferritin protein in the *E. coli* and silkworm expression system can self-assemble into spherical particles, and the Hemagglutinin protein can be attached to the surface of the spherical particles.

Compared with H antigen alone, the H-Fe nanoparticle antigen can induce mice to produce higher levels of specific and protective antibodies.

The fusion protein is not fully folded in *E. coli*. In contrast, due to the post-translational modification function of silkworm eukaryotic expression system, the fusion protein can be fully folded, which is more helpful to inducing the body to produce protective antibodies. Silkworm expression system is more suitable for the application of self-assembly ferritin technology than *E. coli* expression system.
