# Supplementary figures and images for: Expression and Evaluation of a Novel PPRV Nanoparticle Antigen Based on Ferritin Self-Assembling Technology
Source: Pharmaceutics. 2022 Sep 8;14(9):1902. doi: 10.3390/pharmaceutics14091902 (PMC9500948; doi:10.3390/pharmaceutics14091902)

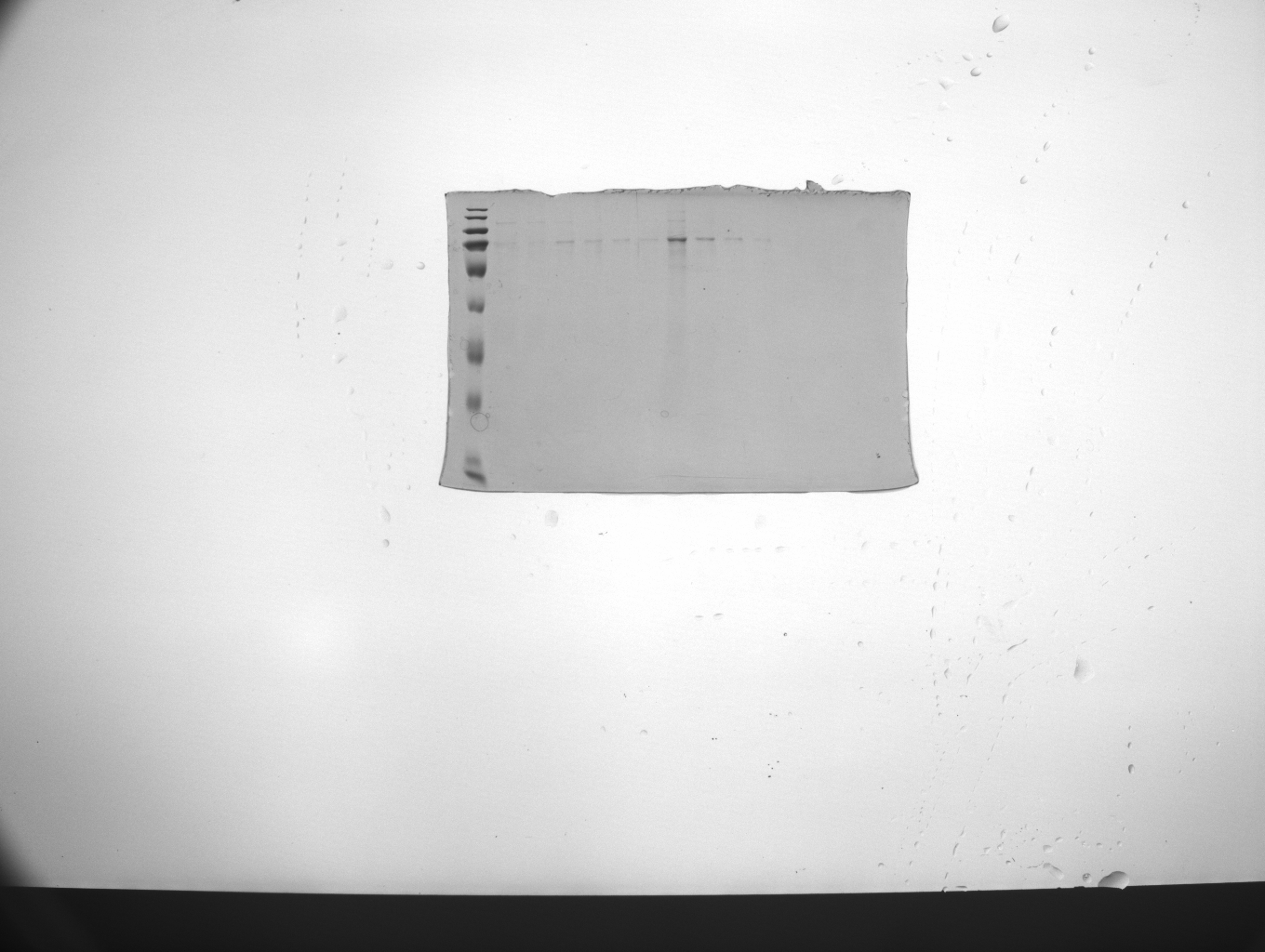

Supplement: Supplementary file 1 [file pharmaceutics-14-01902-s001.zip › Figure S1A.tif]

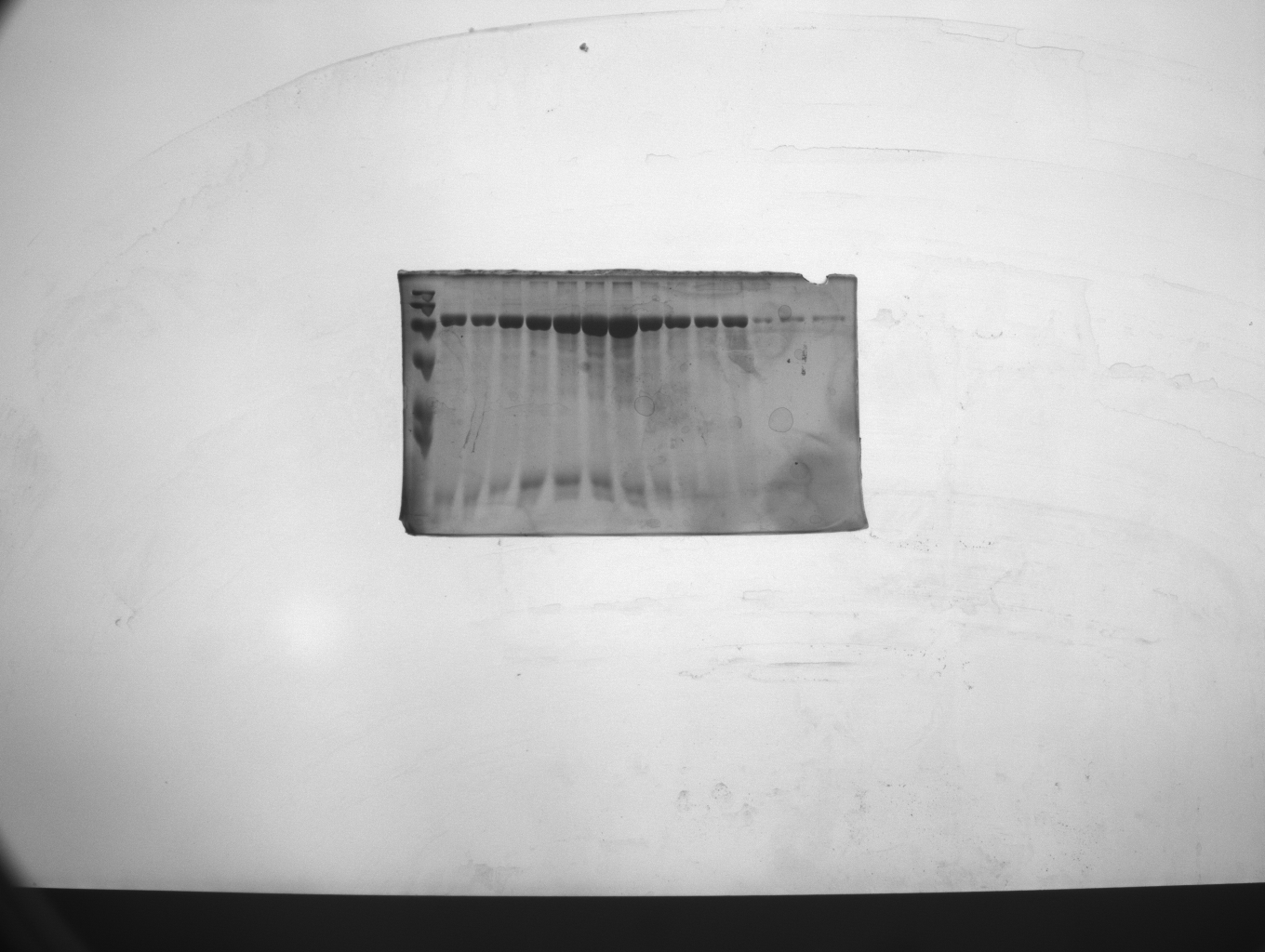

Supplement: Supplementary file 1 [file pharmaceutics-14-01902-s001.zip › Figure S1B.tif]
